# Resolving taxonomic confusion: establishing the genus *Phytobacter* on the list of clinically relevant Enterobacteriaceae

**DOI:** 10.1007/s10096-022-04413-8

**Published:** 2022-02-15

**Authors:** Theo H. M. Smits, Lavinia N. V. S. Arend, Sofia Cardew, Erika Tång-Hallbäck, Marcelo T. Mira, Edward R. B. Moore, Jorge L. M. Sampaio, Fabio Rezzonico, Marcelo Pillonetto

**Affiliations:** 1 Environmental Genomics and Systems Biology Research Group, Institute of Natural Resource Sciences (IUNR), Zurich University of Applied Sciences ZHAW, Wädenswil, Switzerland; 2 Central Public Health Laboratory – State of Paraná – LACEN/PR, Molecular Bacteriology Division, São José Dos Pinhais, PR Brazil; 3 Culture Collection University of Gothenburg (CCUG), Department of Clinical Microbiology, Sahlgrenska University Hospital, Region Västra Götaland, Gothenburg, Sweden; 4 Department of Clinical Microbiology, Sahlgrenska University Hospital, Region Västra Götaland, Gothenburg, Sweden; 5 Core for Advanced Molecular Investigation, Graduate Program in Health Sciences, School of Medicine, Pontifícia Universidade Católica Do Paraná, Curitiba, PR Brazil; 6 Department of Infectious Disease, Institute for Biomedicine, Sahlgrenska Academy, University of Gothenburg, Gothenburg, Sweden; 7 Faculdade de Ciências Farmacêuticas - University of São Paulo and Fleury Medicina Diagnóstica, São Paulo, SP Brazil

**Keywords:** *Phytobacter diazotrophicus*, *Phytobacter ursingii*, *Phytobacter palmae*, Genomics, Identification, Taxonomy

## Abstract

Although many clinically significant strains belonging to the family *Enterobacteriaceae* fall into a restricted number of genera and species, there is still a substantial number of isolates that elude this classification and for which proper identification remains challenging. With the current improvements in the field of genomics, it is not only possible to generate high-quality data to accurately identify individual nosocomial isolates at the species level and understand their pathogenic potential but also to analyse retrospectively the genome sequence databases to identify past recurrences of a specific organism, particularly those originally published under an incorrect or outdated taxonomy. We propose a general use of this approach to classify further clinically relevant taxa, i.e., *Phytobacter* spp., that have so far gone unrecognised due to unsatisfactory identification procedures in clinical diagnostics. Here, we present a genomics and literature-based approach to establish the importance of the genus *Phytobacter* as a clinically relevant member of the *Enterobacteriaceae* family.

## Introduction

The taxonomy of *Enterobacteriaceae*, since its establishment and valid publication in the Approved Lists of Bacterial Names in 1980 [1], has been prone to constant updates, revisions and corrections [2, 3]. Over the years, many novel genus and species names have been validly published. Since about 1985, the use of improved molecular tools such as PCR, 16S rRNA gene and multi-locus sequence analysis (MLSA) has led to a more stable taxonomy [4]. It has been only in the last 10 years that genome-based taxonomic studies and the use of average nucleotide identities (ANI) [5] and digital DNA-DNA hybridization (dDDH) [6] have enabled reliable delineation and detailed analysis of the different taxa, at the species-level, and giving rise in 2016 to the revision of the *Enterobacteriaceae*, with the creation of several sister families, like the *Erwiniaceae* or *Pectobacteriaceae* [2]. A more detailed analysis has shown six distinct phylogenomic-based clades within the family *Enterobacteriaceae *sensu* strictu* [3]. Still, some *Enterobacteriaceae incertae sedis* persist, which cannot be classified in the current taxonomy of the family, while others are not included in such studies.

Even though some medical journals, such as the Journal of Clinical Microbiology and Diagnostic Microbiology and Infectious Diseases, publish regular “taxonomic updates” for their readers [7–12], it may not be so easy for clinical microbiologists to follow all updates systematically. Genome sequencing has greatly accelerated the discovery of new taxa and the need for rearranging old taxonomic relationships [13]. A problem in this perspective is to extract the relevance of the taxonomic changes for clinical microbiologists. A taxonomic change may not be immediately recognised as therapeutically relevant if, due to that taxonomic change, the treatment administered to the patient must not be adapted [14]. On the other hand, the use of outdated taxonomy and misidentification of clinical isolates could prevent the recognition of novel emerging pathogens, cause outbreaks to be overseen or species to be incorrectly held responsible for infections [15]. Furthermore, the deposit of misidentified sequences in the database may, in turn, serve as a seed to propagate errors, causing a cascade effect involving future studies [16].

Most taxonomic confusion can be avoided by critically interpreting the data in nucleotide databases. This requires, however, an additional effort that is often not provided in clinical papers and case reports. Here, we present the case of the genus *Phytobacter* [17, 18] that has emerged from the disentanglement of the former *Erwinia herbicola* – *Enterobacter agglomerans* complex (EEC) [19, 20] and was increasingly detected in clinical settings over the last few years, although many of its isolates are still incorrectly assigned to *Pantoea* spp., *Kluyvera intermedia* or *Metakosakonia* spp. [15, 18, 21–25].

## Genome-based resolution of the genus *Phytobacter*

Clinical isolates belonging to Brenner’s biotype XII had been assigned to the *E. agglomerans* complex [19] after their first detection [26] but were erroneously transferred to *Pantoea agglomerans*, when this species was split off from the *E. agglomerans* clade [27]. They were later recognised to be distinct from *P. agglomerans* after phenotypic and genotypic analyses of that species [16, 28] but remained without a reliable taxonomic identification. Only in 2018, these strains were included in the genus *Phytobacter* [17, 18], when the isolates associated with a multistate outbreak in Brazil, caused by contaminated total parenteral nutrition [29], were found to cluster with isolates of Brenner’s biotype XII [19]. This biotype was one of the last biotypes yet to be assigned to a distinct taxonomic rank among those that were reclassified from the *E. agglomerans* complex [28]. Based on the 16S rRNA gene and MLSA sequence data, the taxonomic positions of earlier clinical isolates belonging to biotype XII [30–32] could thus be revised and assigned to two distinct species: *Phytobacter diazotrophicus* and *Phytobacter ursingii* [24].

**Fig. 1:**
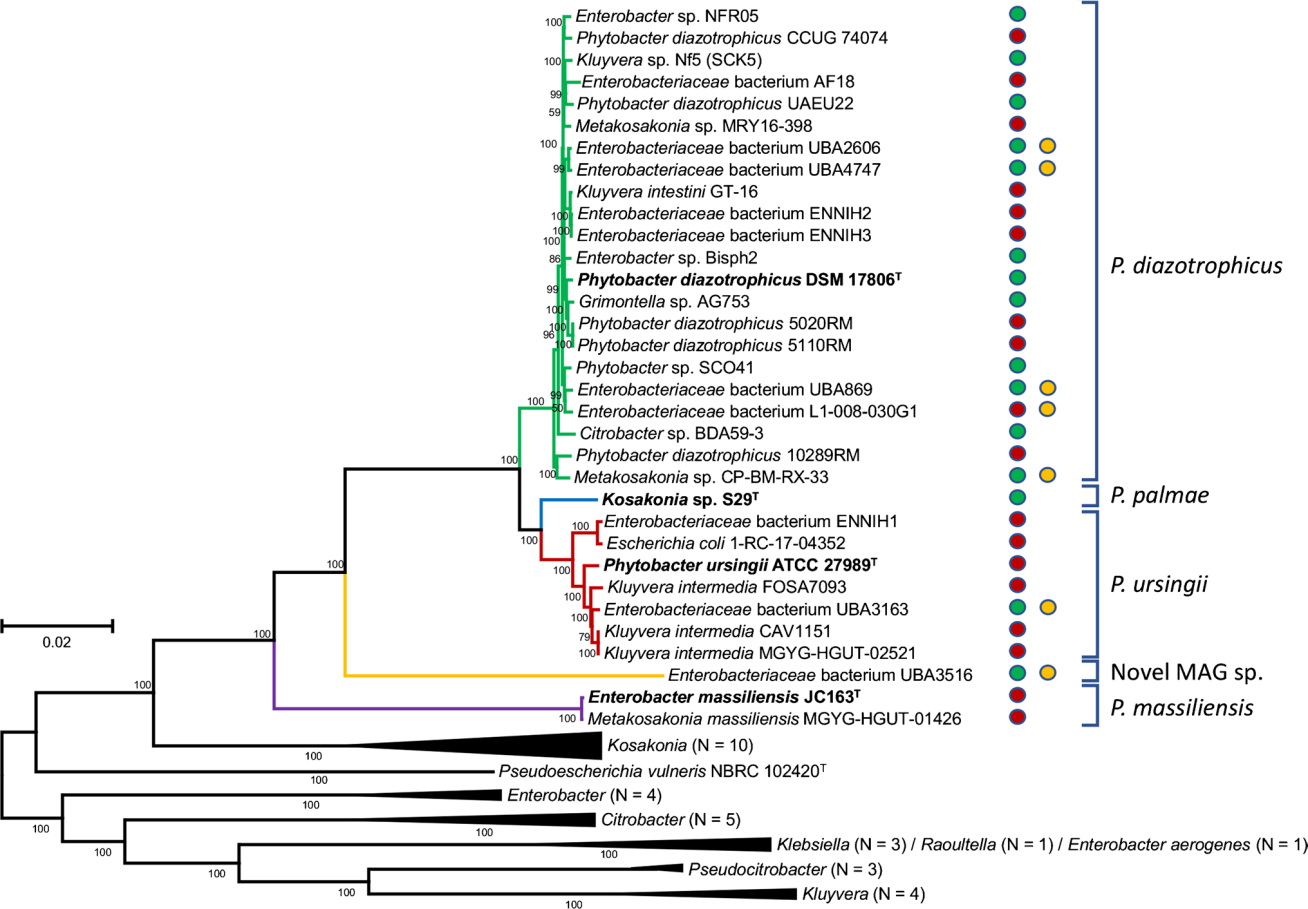
Core-genome tree, generated using EDGAR 3.0, with all available genomes of *Phytobacter* spp. (December 2021). Original descriptions as currently present in GenBank are indicated. Approximately maximum-likelihood phylogenetic trees were generated by aligning all core genes with MUSCLE, concatenation and tree generation with FastTree. The most optimal tree, based on 1424 genes per genome (476,326 amino acids per genome), is shown. Values at the branches are local support values computed by FastTree, using the Shimodaira–Hasegawa test. Line colours represent the different species: green: *P. diazotrophicus*, red: *P. ursingii*, blue: *P. palmae*, yellow: new *Phytobacter* sp.; purple: *P. massiliensis*. Type strains are indicated in bold. The origin of each strain is indicated as a dot: red, clinical; green, environmental; yellow, MAG sequence

Using modern tools based on whole-genome sequencing (WGS), analysis with digital DNA-DNA-hybridization (dDDH) and average nucleotide identities (ANI), the phylogeny of the genus *Phytobacter* has been resolved (Fig. 1, created with EDGAR v. 3.0 [33]). Some of the isolates of a US outbreak involving infusion fluids, initially described as *Pantoea* (*Enterobacter*) *agglomerans* during the early 1970s, could be identified as *P. diazotrophicus* (ATCC 27981 and ATCC 27990) and *P. ursingii* (ATCC 27982 and ATCC 27989^ T^) [24, 30–32] within the framework of the investigation of the 2013 Brazilian outbreak. A third species of the genus, *Phytobacter palmae*, was then identified from oil palm (*Elaeis guineensis*) in Malaysia [34], while a fourth species, also isolated from the bloodstream of Brazilian patients, will soon be described (M. Pillonetto, unpublished results).

Additionally, strain JC163^T^, originally isolated as *Enterobacter massiliensis* [35] and later renamed *Metakosakonia massiliensis* [3], was recently proposed as *Phytobacter massiliensis* based on genomic comparisons [18]. Rule 38 of the International Code of Nomenclature of Prokaryotes [36] recalls the priority of publication, which gives, in this case, the genus name *Phytobacter* priority over the genus name *Metakosakonia* [37]. A metagenome-assembled genome (MAG) currently assigned as *Enterobacteriaceae* bacterium UBA3516 may belong to another novel species of the genus *Phytobacter* [38] (Fig. 1). However, both genomes of *P. massiliensis* and that of *Enterobacteriaceae* bacterium UBA3516 branch deeper in the core-genome tree and do not contain the nitrogen fixation gene cluster (*nif-*genes), which are described as a key feature of the genus *Phytobacter*.

Even though the taxonomy of the genus *Phytobacter* has been well defined in the last few years, many genomes that can be retrieved from GenBank and can unambiguously be assigned to the genus *Phytobacter* based on the ANI analysis [24] (Table 1), are deposited therein under different names (Fig. 1). The range of potential genus names that may hide misidentified *Phytobacter* isolates include *Metakosakonia*, *Kluyvera*, *Enterobacter*, *Pantoea*, *Citrobacter*, *Enterobacteriaceae* bacterium, or “*Grimontella*”. The genus “*Grimontella*” was never validly published, although a 16S rRNA gene sequence of “*Grimontella senegalensis*” strain C1p was deposited at NCBI (accession number AY217653). As the name is not validly published, it has no standing in taxonomy. The other names were based on the misidentification of “*Kluyvera intermedia*” CAV1151 (now *P. ursingii* CAV1151) [24, 39] and the genome-based phylogenetic analysis of “*E. massiliensis*” JC163 [35]. This species was, meanwhile, renamed “*M. massiliensis*” [3]. In the same publication, the strain “*Kluyvera intestini*” GT-16 was discussed to be a *Metakosakonia* sp. as well [3]. The strain GT-16 is here included within *P. diazotrophicus* (Table 1; Fig. 1)[40], whereas *M. massiliensis* was, based on ANI values, included in the genus *Phytobacter* as *P. massiliensis* [18].

**Table 1:** Currently available genomes of *Phytobacter* strains (December 2021) as extracted from NCBI GenBank. The whole-genome-based core phylogeny of these strains is shown in Fig. 1. The name under which the strain was originally or intermediary described is given between double quotation marks in the second column

Strain	Former/Misidentified name	Source	Source type^1^	Origin	Year	MDR genes^2^	Reference
*Phytobacter diazotrophicus*							
DSM 17806^ T^		Wild rice (*Oryza rufipogon*)	E	People’s Republic of China, Lingshui	2004	None	[17]
5110RM		Total parenteral nutrition	H	Brazil, Curitiba	2013	None	[24]
5020RM		Human, blood	H	Brazil, Curitiba	2013	None	[24]
10289RM		Human, rectal swab	H	Brazil, Curitiba	2015	*bla*_KPC_	[24]
Bisph2	“*Enterobacter*” sp.	Sandy soil	E	Algeria, Biskra	2012	ND^2^	[41]
ENNIH2	“*Enterobacteriaceae*” bacterium	Hospital wastewater	H	USA, MD	2012–2016	*bla*_KPC_	[42]
ENNIH3	“*Enterobacteriaceae*” bacterium	Hospital wastewater	H	USA, MD	2012–2016	*bla*_KPC_	[42]
GT-16	“*Kluyvera intestini*”	Human, stomach	H	USA	2015	*bla*	[21, 43]
SCO41	*Phytobacter* sp.	Gut of *C. elegans*	E	People’s Republic of China	2013	ND	[44]
MRY16-398	“*Metakosakonia*” sp.	Human, sigmoid colon diverticulitis	H	Japan	2015	*bla*_IMP—6_	[23]
AG753	“*Grimontella*” sp.	Rice bacterial endophyte	E	Italy	2011	ND	Unpublished
Nf5 (SCK5)	“*Kluyvera*” sp.	Plant growth promoting bacterium from sugarcane	E	Colombia, Valle del Cauca	2014	ND	[45]
NFR05	“*Enterobacter*” sp.	Bacterial root endophyte of switchgrass	E	USA	NI^2^	ND	Unpublished
AF18		Bile sample, coinfection with *K. pneumoniae*	H	People’s Republic of China, Peking	2020	*bla*_CTX-M3_	[46]
UAEU22		Rhizosphere of date palm	E	Ras Al Khaimah, United Arab Emirates	2019	ND	[47]
BDA59-3	“*Citrobacter*” sp.	Rice leaves	E	Italy	2019	ND	[48]
CCUG 74074		Blood	H	Sweden	2019	ND	Unpublished
UBA869	“*Enterobacteriaceae*” bacterium	MAG^2^, terrestrial metagenome	E	USA, New York City	NI	ND	[38]
UBA2606	“*Enterobacteriaceae*” bacterium	MAG, terrestrial metagenome	E	USA, New York City	NI	ND	[38]
UBA4747	“*Enterobacteriaceae*” bacterium	MAG, terrestrial metagenome	E	USA, New York City	NI	ND	[38]
L1-008-030G1	“*Enterobacteriaceae*” bacterium	MAG, infant faeces, human gut metagenome	H	USA, Pittsburgh	2016/2019	ND	Unpublished
CP_BM_RX_33	*“Metakosakonia”* sp.	MAG, rhizosphere of *Barbacenia macrantha*	E	Brazil, Minas Gerais	2017	ND	Unpublished
*Phytobacter ursingii*							
ATCC 27989^ T^	“*Enterobacter agglomerans*” “*Pantoea agglomerans*”	Human, sputum	H	USA, SC	1974	None	[24, 30]
CAV1151	“*Kluyvera intermedia*”	Human, perirectal	H	USA, VA	2009	*bla*_KPC_	[39]
ENNIH1	“*Enterobacteriaceae*” bacterium	Hospital wastewater	H	USA, MD	2012–2016	*bla*_KPC_	[42]
FOSA7093	“*Kluyvera intermedia*”	Intraabdominal pancreas cyst	H	Denmark, Copenhagen	2016	*bla*_CTX-M-type_	[22]
1-RC-17–04352	“*Escherichia coli*”	Sink	H	USA, Milwaukee	2017	ND	Unpublished
UBA3136	“*Enterobacteriaceae*” bacterium “*Stenotrophomonas maltophilia*”	MAG, terrestrial metagenome	E	USA, New York City	NI	ND	[38]
UHGG_MGYG-HGUT-02521	“*Kluyvera intermedia*”	Human gut	H	NI	NI	*bla*_KPC_	[49]
*Phytobacter palmae*							
S29^T^	“*Kosakonia*” sp.	Oil palm	E	Malaysia	2012	ND	[34]
*Phytobacter massiliensis*							
JC163^T^	“*Enterobacter massiliensis*””*Metakosakonia massiliensis*”	Human, stool sample	H	Senegal	2011	ND	[2, 18, 35]
MGYG-HGUT-01426	“*Metakosakonia massiliensis*”	Human gut	H	NI	NI	ND	[49]
Novel MAG species							
UBA3516	“*Enterobacteriaceae*” bacterium	MAG, terrestrial metagenome	E	USA, New York City	NI	ND	[38]

^1^E: environmental isolate; H: hospital-associated isolate

^2^Abbreviations: *MDR*, multidrug resistance; *ND*, not determined; *NI*, no indications; *MAG*, metagenome-assembled genome

The emergence of members of the genus *Phytobacter* under contradicting names in different publications over the past years [18, 24, 25] creates additional problems in handling the genome sequence database (Fig. 1), as NCBI refuses to rename the organisms and/or the phylogenetic assignment without the formal approval of the original submitters. Therefore, improving genomic-based taxonomy will remain a challenge that can currently be tackled only by submitting a sufficient number of genomes with the correct genus and species names.

## Misidentifications in clinical laboratories

Unfortunately, isolates belonging to the various Brenner’s biotypes were probably among those used to generate the phenotypic profiles that are the basis for identification of *P. agglomerans*, by the BD Phoenix™ diagnostic system, thus leading to erroneous results when performing routine identifications, using the corresponding biochemical panels [16]. The same results (i.e., *Phytobacter* spp., misidentified as *Pantoea* sp. or *P. agglomerans*) were obtained when other automated systems, such as Vitek® 2 (bioMérieux) or Microscan® (Beckman Coulter) or manual methods, such as API 20E® (bioMérieux), were used in the laboratories [29]. This indicates that the common phenotypic identification methods in clinical laboratories cannot distinguish *Phytobacter* isolates from *Pantoea* spp., without additional effort.

Based on the data available in the literature, we have identified more instances in which *Phytobacter* may have been potentially misidentified as *P. agglomerans* (Table 2) and that resulted in the overestimation of the role of the latter species as an opportunistic human pathogen [15, 50]. The case of Boszczowski et al. [51] is emblematic of how erroneously assigned sequences in databases or misnamed catalogue entries can be detrimental for future identifications if they are not carried out with proper diligence. Using the *gyrB* sequence of their isolate, the authors identified it as *P. agglomerans*, based on the 100% BLASTN match with strain ATCC 27990, which was then still listed as *P. agglomerans* in the ATCC catalogue. Strain ATCC 27990, however, had been already excluded from *P. agglomerans* by previous taxonomic studies [15, 28], and the corresponding *gyrB* sequence in NCBI was, at that time, tentatively listed as *Enterobacter* spp. The proper line of action by the authors would have been a comparison of their sequence, not simply with just the first BLAST match, but with a number of the type strains of potential matching species, using clustering analysis. Only later, strain ATCC 27990 was unequivocally identified as a member of the species *P. diazotrophicus* [24], a fact that was confirmed by genome sequencing. Meanwhile, the name of the strain was also corrected on the ATCC website and in the corresponding NCBI entries, together with the name of three further strains assigned initially to Brenner’s biotype XII, i.e. ATCC 27981 (*P. diazotrophicus*) and ATCC 27982 and ATCC 27989 (*P. ursingii*).

**Table 2:** Misidentification of cases and outbreaks potentially including *Phytobacter* spp

Case study^1^	Number of cases	Identification method	Supposed species	Status^2^	Reference
Clinical	6	Biochemical	*Enterobacter* spp.	Some ATCC-deposited strains confirmed *P. diazotrophicus* or *P. ursingii*	[30]
NICU, TPN	8	API-20E	*Pantoea* spp*.*	Open	[52]
Nosocomial sepsis	6	Vitek-2, GNI card	*P. agglomerans*	Open	[53]
Nosocomial sepsis	6	Vitek, GN card	*P. agglomerans*^3^	Open	[54]
Contaminated TPN	8	Vitek-2, API-20E, *gyrB*	*P. agglomerans*	Confirmed *P. diazotrophicus*	[51]
Neonatal sepsis	1	Vitek-2, GN25 card	*Pantoea* spp.	Open	[55]
NICU, septicaemia	14	API-20E	*P. agglomerans*	Open	[56]
Bloodstream infection	12	Vitek-2, PFGE	*P. agglomerans*	Open	[57]
Clinical samples	40	Biochemical, Vitek-2	*P. agglomerans*	Open	[58]
Paediatric patients	14	BD Crystal, MALDI-TOF MS	*P. agglomerans*	Open	[59]

^1^Abbreviations: *NICU*, neonatal intensive care unit; *TPN*, total parenteral nutrition; *PFGE*, pulsed-field gel electrophoresis; *MALDI-TOF MS*, matrix-assisted laser desorption-ionisation time-of-flight mass spectrometry

^2^Interpretation of the data by the authors of this manuscript

^3^*Rahnella aquatilis* and *Candida famata* co-isolated in one sample

## Useful microbiological features for the correct identification of *Phytobacter* spp.

*Phytobacter* spp. should be suspected when a strong lactose-fermenting Gram-negative colony is isolated from human samples on MacConkey Agar. The colony morphology on this medium resembles that of *E. coli* and/or *Citrobacter*, having a variable phenotype [60]. Sometimes strains will show the classic bile salt halo-surrounding colonies. Some strains of *P. diazotrophicus* and *P. ursingii* are variably strong lactose-fermenters. Additionally, it can yield lactose-negative or even weak lactose-positive colonies, the latter having a colony morphology that resembles strains belonging to the *Enterobacter cloacae* complex. Differently from most true *P. agglomerans* isolates and related species [27, 61, 62], *Phytobacter* strains do not produce a yellow pigment [24].

Biochemical tests with *Phytobacter* spp. display a typical profile (Table 3) giving triple-negative results for lysine decarboxylase, arginine dihydrolase, and ornithine decarboxylase [24]. These features generally rule out *E. coli* and *C. amalonaticus* (Table 3). In the commercial identification tables and systems, this may lead to confusion with *Pantoea* spp. [16], but *Phytobacter* is using citrate as a carbon source (Table 3). Furthermore, *Phytobacter* spp. can ferment most of the sugars commonly used in manual and automated systems for bacterial identification, except for inositol and melibiose.

**Table 3:**
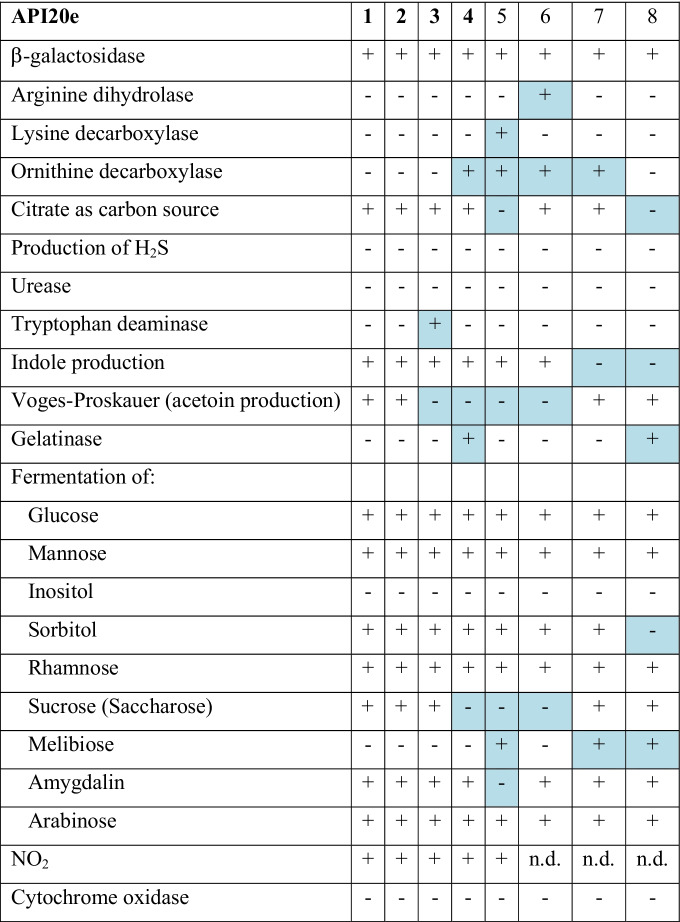
Phenotypic characterization of *Phytobacter* spp. (columns 1-4, bold) and related organisms as reported by API20e test strips. 1: *Phytobacter diazotrophicus* (*n* = 14); 2: *Phytobacter ursingii* (*n* = 6); 3: *Phytobacter palmae* (*n* = 1); 4: *Phytobacter massiliensis* JC163^T^; 5: *Escherichia coli* DSM 30083^ T^; 6: *Citrobacter amalonaticus* CCUG 4860^ T^ 7: *Kluyvera intermedia* DSM 4581^ T^; 8: *Pantoea agglomerans* ATCC 27155^ T^. Data from own experiments (1–3) or from BacDive (https://bacdive.dsmz.de/) (4–8)). Differences from the majority are highlighted in blue

Clinical microbiologists should be aware that, in the absence of a regularly updated reference database, mass spectrometry systems (MALDI-TOF MS, Vitek-MS, and MALDI Biotyper-Bruker Microflex) can generate a wide choice of false identifications for strains of *Phytobacter* spp. The erroneous output can include different *Pantoea* spp., *Leclercia adecarboxylata, Pseudescherichia vulneris, Klebsiella ozaenae, Klebsiella oxytoca, Enterobacter cloacae, Salmonella* spp., or even indicate as “unidentified species”. Such outputs are not convincing and may further add to the discrepancies already found in the literature [63]. An alternative used in our labs is to implement an in-house SuperSpectrum for the MALDI-TOF MS identification of *Phytobacter* spp. (M. Pillonetto, unpublished results). Following this strategy, sixteen suspicious strains were identified in Brazil between 2016 and March 2021 as *Phytobacter* spp. and ultimately confirmed by WGS (Table 4).

**Table 4:** Recently isolated clinical strains of *Phytobacter* spp. not yet published

Country	City, state	Species	Number of isolates	Isolation period	Sample(s), (number of isolates)	MDR^1^-positive	MDR-negative
Switzerland	Zürich^2^	*P. diazotrophicus*	2	2013–2014	Blood (2)	0	2
Sweden	Gothenburg, Kalmar, Uppsala	*P. diazotrophicus*	5	2008–2020	Urine (1), blood (2), CAPD-fluid (1), sputum (1)	NA^1^	NA
	Gothenburg	*P. ursingii*	1	2015	Blood (1)	NA	NA
Brazil	Curitiba-PR, São Paulo-SP, Cuiabá-MT	*P. diazotrophicus*	9	2016–2021	Urine (1), blood (4), rectal swab (2), eye infection (1), tracheal aspirate (1)	3	6
	Curitiba-PR, São Paulo-SP	*P. ursingii*	5	2016–2020	Urine (1), blood (2), catheter (1), rectal swab (1)	1	4
	Curitiba-PR	Novel species	2	2020	Blood (2)	0	2

^1^Abbreviations: *MDR*, multidrug-resistant; *NA*: not analysed

^2^Source: Dr. Frank Imkamp University of Zürich, Institute of Medical Microbiology, Molecular Diagnostics group, Zürich, Switzerland

**Fig. 2:**
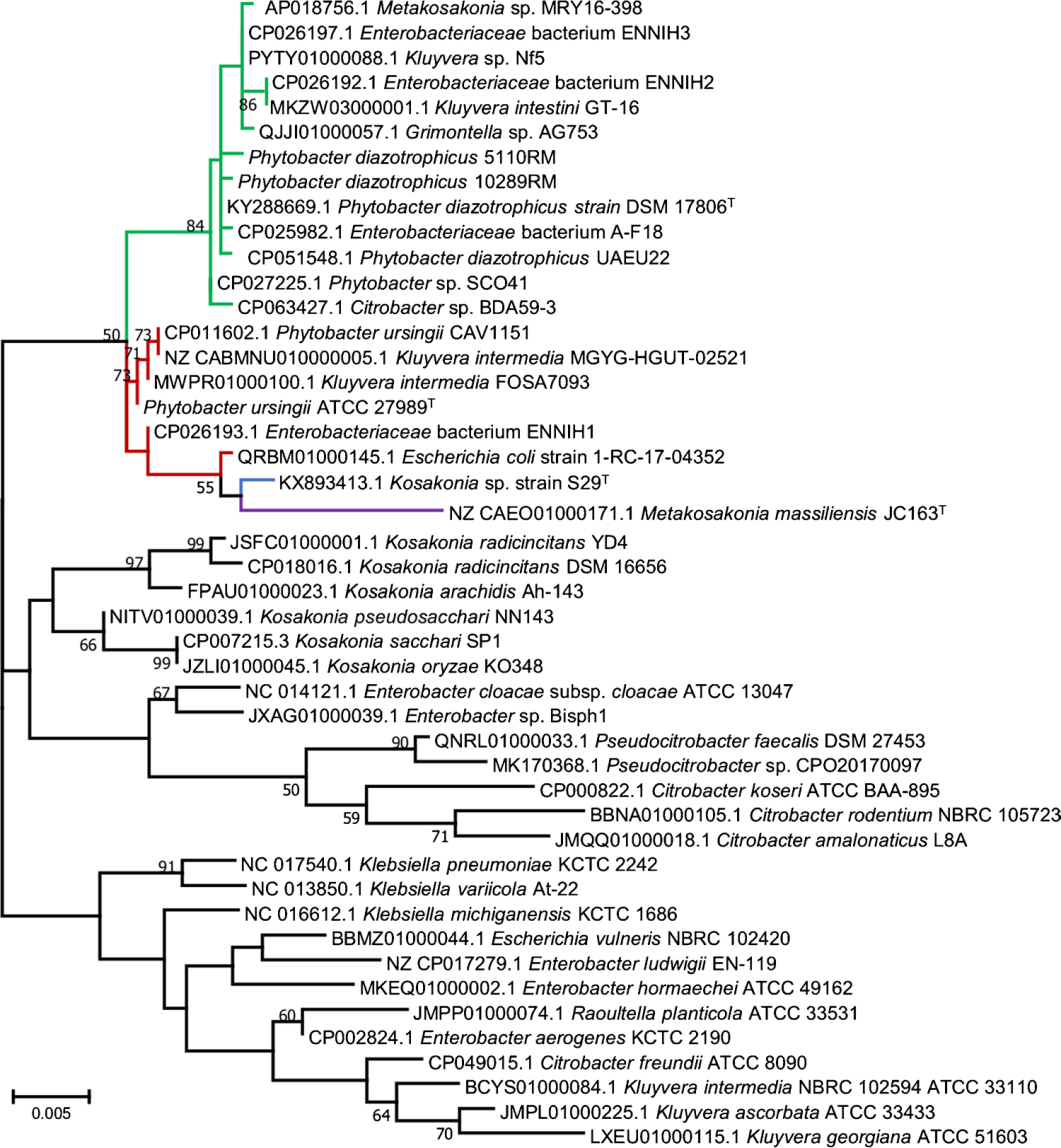
Phylogenetic tree showing the evolutionary relationship among *Phytobacter* species and other type species of the family *Enterobacteriaceae* based on 16S rRNA gene sequences (1457 bp). The tree was constructed by using the maximum likelihood method and the most optimal tree is shown. Line colours represent the different species: green: *P. diazotrophicus*, red: *P. ursingii*, blue: *P. palmae*, purple: *P. massiliensis*. Numbers at branching points are bootstrap percentage values (> 50%) based on 1000 replications. GenBank accession numbers are shown before the strain name. Bar, 0.005% nucleotide sequence difference

The most optimal solution to avoid this problem is for uncertain isolates to be further identified, using sequencing of the 16S rRNA gene (Fig. 2) or, preferably, the *gyrB* gene, as the housekeeping gene is known to enable good resolution within the *Enterobacteriaceae* [15, 28]. If the investigation is relevant (as in an outbreak) and access to the technology is available, whole-genome next-generation sequencing (NGS) is the most reliable approach to identify the suspect isolates to the species level, using ANI or dDDH or core-genome phylogeny.

The clinically oriented Culture Collection University of Gothenburg (CCUG) in Sweden has archived six isolates of *P. diazotrophicus* and two isolates of *P. ursingii* in its collection, all isolated in clinical samples in Sweden since 2012. These are presently identified based on 16S rRNA gene sequence and *dnaJ* gene sequence [64], which also work well for *Enterobacteriaceae*. The gene sequence allows for clear discrimination from other species and genera. The whole-genome sequence of isolate *P. diazotrophicus* strain CCUG 74074 corresponded well within the core-genome phylogeny of the genus (Fig. 1). Therefore, the *dnaJ* gene may be well-suited for fast and concise identifications of the members of the genus, as well.

## Clinical relevance

Although *Phytobacter* has been described in human samples only recently [24], it has unequivocally been proved by WGS that it has previously been misidentified as other species, such as *Pantoea* or *Kluyvera*, since the 1970s [18, 24, 25, 46]. In the last 5 years, *Phytobacter* has been described in important clinical samples such as blood, sputum, digestive tract, and bile (Table 1). We reported 24 human isolates in a 5-year period (2016–2021), including thirteen from the bloodstream and one from a catheter, reinforcing the concept that the genera *Phytobacter* is clinically relevant (Table 4). Another indirect support is the large number of publications referring to outbreaks or case reports of *Pantoea agglomerans* and *Pantoea* spp. infection (Table 2), wherein the organisms in question were poorly identified by manual or automated tests only, which could easily lead to the misidentification of isolates potentially belonging to the genus *Phytobacter*, as already observed by our research groups [16, 24].

The first published isolates of *Phytobacter* were sensitive to common antibiotics [24, 31]. However, in recent years, the number of multidrug-resistant *Phytobacter* isolates has increased. Several isolates for which genomes are available (Table 1, Table 4) show that multidrug resistance is present, including critical antimicrobial resistance genes such as *bla*_KPC_, *bla*_IMP6_, and *bla*_CTX-M_ [18, 23, 29, 39, 43, 46]. Genomic analysis has revealed that in many cases, resistance is encoded on plasmids [42], thus potentially transferred from other hospital-related pathogens. It may be possible that *Phytobacter* spp. have adapted from their natural ecological niches as soil or plant-associated bacteria to the hospital environment and can easily exchange resistance plasmids or other mobile genetic elements, potentially becoming a new, emerging threat as a multidrug-resistant microorganism.

## Future directions

**Fig. 3:**
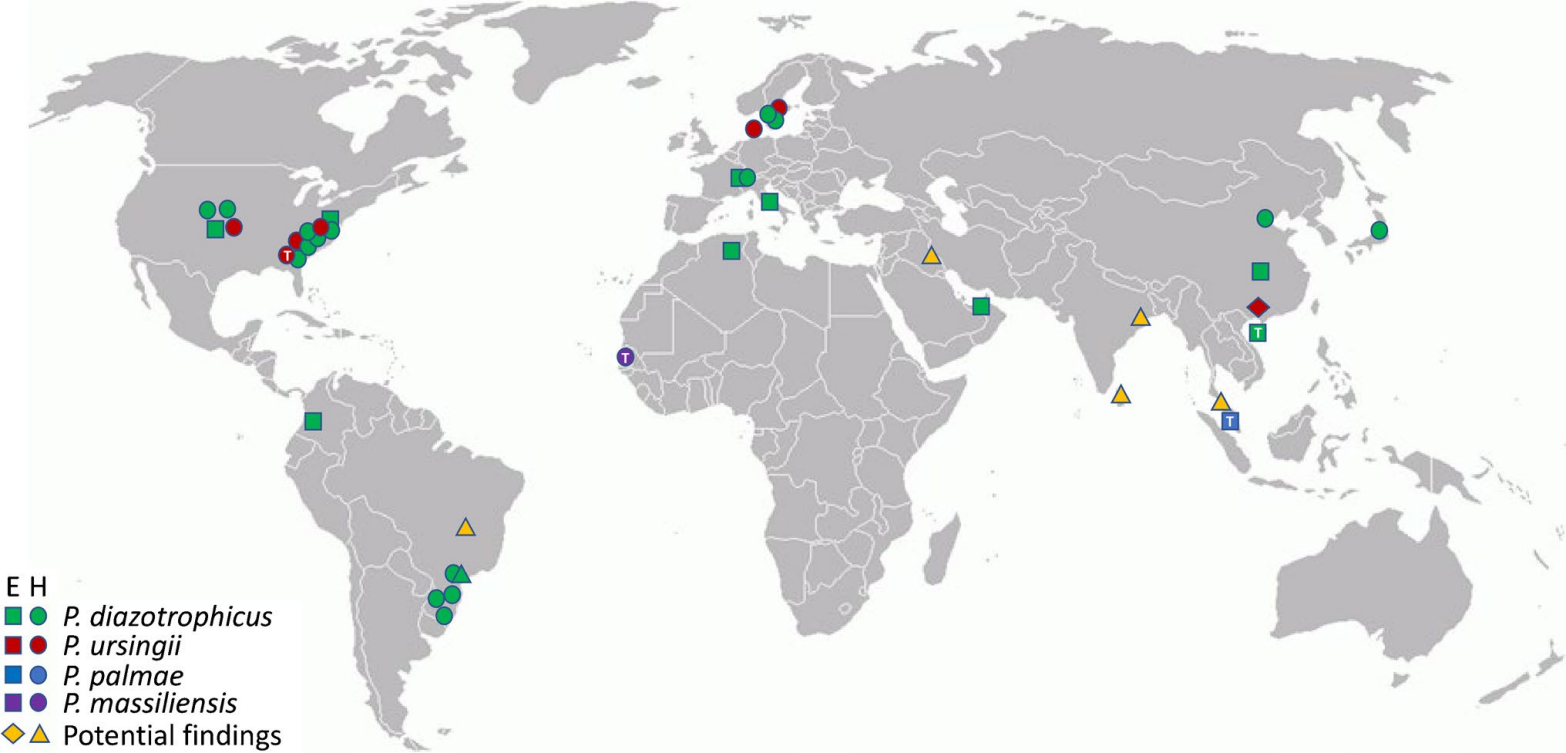
Geographic overview of *Phytobacter* spp. distribution. Coloured squares and circles represent the different species for which genomes are available (Table 1): green: *P. diazotrophicus*, red: *P. ursingii*, blue: *P. palmae*, violet: *P. massiliensis*. A white “T” in the symbol represents the location of the type strain. Diamonds and triangles (orange) indicate the origins of potential findings as reported in Table 2. E: environmental isolate; H: hospital-associated isolate

The clinical history of *Phytobacter*, with outbreaks mainly on the US East Coast and in Brazil [29, 31, 51] but also with individual cases at other locations worldwide (Fig. 3), shows us that clinicians should be(-come) aware of the importance of this genus as a recurring opportunistic pathogen. The clinical relevance has increased over the last few years, although it is not clear from the literature whether more cases could have been ascribed to this organism. Many of the current papers about clinical infections in humans caused by emerging and/or opportunistic *Enterobacterales*, such as *Pantoea* spp., *Kluyvera* spp., *Raoultella* spp., and *L. adecarboxylata* are attributing the identifications of the pathogen to the species-level, using only intrinsically inaccurate phenotypic-based methods, such as manual (API-20E) or automated biochemical profiling (Vitek-2, Phoenix, or Walkaway systems). However, the major problem is that the databases on which these commercial platforms rely are commonly outdated. Their updates involve prolonged and complex verification and certification processes, which does not keep up with the pace of developments in bacterial taxonomy. Erroneous designations can impair outbreak investigation and compromise epidemiological studies of etiological agents, especially in hospital-acquired infections. In this sense, correct identifications of *Phytobacter* remain challenging for routine clinical laboratories. Clinical microbiologists need to be aware of this and other new species that are becoming increasingly relevant to infectious diseases [11].

Many of the cases identified as *Phytobacter* spp. infection consisted of sepsis after receiving intravenous fluids or the use of medical devices, often in neonatal intensive care units (Table 2). Neonates may not yet be able to deal with this organism due to a still-developing immune system, whereas infants and adults respond better to infection. Outbreaks of *Phytobacter* spp. have already been shown to be able to lead to high mortality of mainly young and immunocompromised patients [32, 51, 52]. The coinfection of *Phytobacter* spp. with other potential pathogens can result in clinical situations that are even more complicated [29, 46]. In our previous study, combinations of three bacteria (*P. diazotrophicus*, *Acinetobacter baumannii*, and *Rhizobium radiobacter*) were often found in clinical outbreak samples, while one sample contained all three species at once [29]. A recent study showed coinfection of *P. diazotrophicus* with *Klebsiella pneumoniae* with the latter bacterium acting as the major pathogen but being protected against antibiotics by a resistant strain of *P. diazotrophicus* [46]. These examples of bacterial coexistence should be followed in more detail in clinical diagnostics, as they appear to be more common than expected.

## Conclusion

Although the currently reported number of *Phytobacter* infections is not very high, the clinical relevance of this organism may actually be masked by inadequate identification procedures. We understand the impracticality of integrating additional steps in routine clinical diagnostics, but in order to preserve scientific integrity and avoid detrimental taxonomic confusion, it is critical for the most accurate available identification approach to be applied at least in those cases that are to be published in the scientific literature. This is especially important because clinical samples are rarely retained for subsequent independent verification after initial analysis [50]. Based on our investigations, *Phytobacter* potentially may have the same (or even a greater) clinical relevance as *Kluyvera* spp. or *Pantoea* spp., especially since isolates are often confused with species of those genera [24, 42, 43] (Tables 1 and 2). An appropriate identification protocol that targets a reference gene or the genome sequence would be required to better understand the occurrence of *Phytobacter* in clinical samples. Comparison of generated sequences to curated databases such as the Type Strain Genome Server (https://tygs.dsmz.de/), which contains the verified genomes of bacterial type species (including *Phytobacter*), rather than to NCBI, further can contribute to enhancing identification accuracy. On the other hand, the improvement of current biochemical and mass spectrometry methods to include *Phytobacter* spp. in their list of reference organisms is critical for all situations in which sequencing approaches might not be available. Additionally, it is essential to perform proper monitoring of antimicrobial resistance in members of this genus, as the number of cases of multidrug resistant *Phytobacter* is already increasing now.

With the current improvements in the field of genomics [18, 40], it is possible to generate high-quality data to help understand the pathogenic potential of individual nosocomial *Phytobacter* isolates in comparison to that of environmental isolates. Further work will focus on developing molecular diagnostic procedures for field and clinical studies and improving the current databases to resolve the taxonomic confusion so that new isolates can immediately be assigned to the correct genus.

## Data Availability

Genome data were extracted from GenBank or available from the authors after reasonable request.
